# Method for persistent topological features extraction of schizophrenia patients’ electroencephalography signal based on persistent homology

**DOI:** 10.3389/fncom.2022.1024205

**Published:** 2022-10-05

**Authors:** Guangxing Guo, Yanli Zhao, Chenxu Liu, Yongcan Fu, Xinhua Xi, Lizhong Jin, Dongli Shi, Lin Wang, Yonghong Duan, Jie Huang, Shuping Tan, Guimei Yin

**Affiliations:** ^1^College of Geography Science, Taiyuan Normal University, Jinzhong, China; ^2^Institute of Big Data Analysis Technology and Application, Taiyuan Normal University, Jinzhong, China; ^3^College of Resource and Environment, Shanxi Agricultural University, Taigu, China; ^4^Psychiatry Research Center, Beijing Huilongguan Hospital, Peking University Huilongguan Clinical Medical School, Beijing, China; ^5^Laboratory of Data Mining and Machine Learning, College of Computer Science and Technology, Taiyuan Normal University, Jinzhong, China; ^6^College of Applied Science, Taiyuan University of Science and Technology, Taiyuan, China

**Keywords:** topological data analysis, persistent homology, complex brain networks, schizophrenia patients, persistent topological features, EEG signal

## Abstract

With the development of network science and graph theory, brain network research has unique advantages in explaining those mental diseases, the neural mechanism of which is unclear. Additionally, it can provide a new perspective in revealing the pathophysiological mechanism of brain diseases from the system level. The selection of threshold plays an important role in brain networks construction. There are no generally accepted criteria for determining the proper threshold. Therefore, based on the topological data analysis of persistent homology theory, this study developed a multi-scale brain network modeling analysis method, which enables us to quantify various persistent topological features at different scales in a coherent manner. In this method, the Vietoris–Rips filtering algorithm is used to extract dynamic persistent topological features by gradually increasing the threshold in the range of full-scale distances. Subsequently, the persistent topological features are visualized using barcodes and persistence diagrams. Finally, the stability of persistent topological features is analyzed by calculating the Bottleneck distances and Wasserstein distances between the persistence diagrams. Experimental results show that compared with the existing methods, this method can extract the topological features of brain networks more accurately and improves the accuracy of diagnostic and classification. This work not only lays a foundation for exploring the higher-order topology of brain functional networks in schizophrenia patients, but also enhances the modeling ability of complex brain systems to better understand, analyze, and predict their dynamic behaviors.

## Introduction

Topological data analysis (TDA) (Edelsbrunner and Harer, 2010; Ibekwe et al., 2014; Taylor et al., 2015) is related to data analysis, algebraic topology, computational geometry, computer science, and statistics. The main goal of TDA is to use geometry and topology theories to study the qualitative features of data. To achieve this, a precise definition of qualitative features and computational tools in specific practical applications is required. Theories ensure the stability and robustness of these features. One way to achieve this goal is using persistent homology (PH) in TDA (Aktas et al., 2019). Currently, researches on the application of PH to brain network analysis are gaining increasing attention (Lee et al., 2012; Caputi et al., 2021; Xu et al., 2021).

When processing and analyzing brain imaging data, a matrix representing the connection strength between nodes is generated, and a threshold is selected to binarize the matrix. Finally, the adjacency matrix is generated to construct brain networks. The selection of the threshold plays an important role in network construction because it affects the connection density and network topology (Khalid et al., 2014; Chung et al., 2015; Sizemore et al., 2018). Generally, there are three methods for network binarization (Telesford et al., 2011). First, when a connection density is selected as a single threshold, for example, the structure of networks is fully connected when $\frac{2}{N}\,l\,g\,N$ is selected (Castro et al., 2011; Li and Fan, 2013), where *N* is the number of nodes in the networks. This implies that there are no isolated points in the networks. However, this method cannot be applied to real networks because it is suitable only for random networks. The second method involves using a predefined threshold space, wherein the threshold is selected indirectly, and statistical methods are usually use to eliminate the weak connections or pseudo connections. However, the process of threshold selection is complex and not universal when data is changed (Yin et al., 2020; Zhu et al., 2020). Moreover, some important information transmission may be deleted when the weak connections are deleted. The third method involves using a threshold space at condition limits; essentially, the brain network with small-world attributes is built on the selected threshold space. The construction of a random network requires a mean degree of nodes greater than *2lgN* and the same number of nodes and degree of nodes as the original network.

Numerous new thresholding methods have been reported, such as the network’s minimum spanning tree, which builds an unbiased network. Minimum spanning tree is not sensitive to thresholds and density values, so it is considered a good method for network binarization. However, this unbiased network is extremely sparse, which results in several important local connections being ignored (Tewarie et al., 2015). A windowless method based on a thermonuclear Gaussian core has been reported (Huang et al., 2019; Jin et al., 2019). In this method, the false rapid changing states of brain connections in the networks are reduced, and the problem of high-frequency noise is solved when the sliding window method is applied to dynamic brain network analysis.

Although numerous methods have been proposed for selecting a threshold when the brain networks are constructed in different ways, the selection of a network threshold remains difficult because there is no consensus on the best strategy (van Wijk et al., 2010). Therefore, the PH theory in the TDA method was introduced into brain network analysis (Shnier et al., 2019; Caputi et al., 2021). The advantages of this method are that the construction of brain networks does not require binarization, the networks can be analyzed on full-scale, and persistent topological features in the brain networks can be extracted across multiple scales.

## Background

We reviewed some fundamental notions and results from PH that are relevant to our work. For more mathematical introductions, see Zomorodian and Carlsson (2005), Adler et al. (2010), Otter et al. (2017), and Aktas et al. (2019).

### Definition 1

Let a topological space *U* on set *X* be a subset on 2^*X*^, namely, *U*⊂2^*X*^, if the following conditions are met: (1) Φ,*X*⊂*U*; (2) *u*_1_,*u*_2_⊂*U*, *u*_1_∪*u*_2_⊂*U*; (3) *u*_1_,*u*_2_⊂*U*,*u*_1_∩*u*_2_⊂*U*; then, (*X*,*U*) is called the topological space of the finite set *X* (Horak et al., 2009; Edelsbrunner and Harer, 2010).

### Definition 2

In the *n*-dimensional vector space *R^n^* of the real number field, there are a set of vectors *a*_0_,*a*_1_,*a*_2_,⋯,*a*_*n*_, which make {*a*_1_−*a*_0_,*a*_2_−*a*_0_,⋯,*a*_*n*_−*a*_0_} linearly independent. We set up E = θ_0_*a*_0_ + θ_1_*a*_1_ + ⋯ + θ_*n*_*a*_*n*_|θ_0_ + θ_1_ + ⋯ + θ_*n*_ = 1,θ_*i*_ > 0, and the point set E is called an *n*-dimensional simplex (Horak et al., 2009).

A zero-dimensional simplex is a point, a one-dimensional simplex is a line segment, a two-dimensional simplex is a triangle, and a three-dimensional simplex is a three-dimensional triangle.

### Definition 3

Let K be a finite set of simplexes, if the following conditions are met: (1) If σ ∈ *K*, then any face of any simplex in K still belongs to *K*. (2) For σ_1_,σ_2_ ∈ *K*, if σ_1_∩σ_2_ is an empty set or σ_1_∩σ_2_ is on the common side of σ_*1*_ and σ_*2*_, then K is called a simple complex (Horak et al., 2009). The maximum dimension of a simplex in simplex *K* is called the dimension of *K*, expressed as


dimK∈m⁢a⁢x⁢{dim⁢σ}

### Definition 4

For a point cloud aggregation *X*, let *d*(,) represent the distance between two points in a point cloud set. *ℛ*(*X*,ε) is a Vietoris–Rips (VIPs) complex (Carlsson, 2009) if and only if its *k*-dimensional simplex [*x*_0_,*x*_1_,⋯,*x*_*k*_] satisfies *d*(*x*_*i*_,*x*_*j*_)≤ε, 0≤*i*,*j*≤*k*.

## Full-scale brain network analysis model based on PH

**FIGURE 1:**
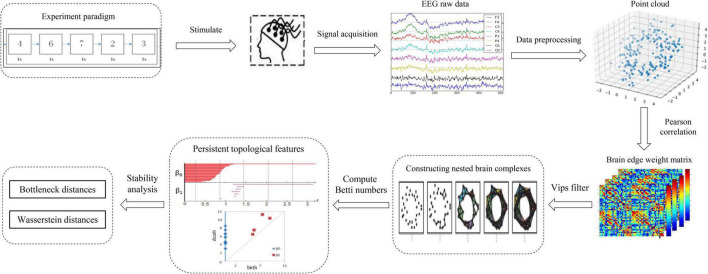
Framework of the full-scale brain network analysis model based on PH.

The full-scale brain network analysis model designed in this study according to the PH data analysis method and the features of electroencephalography (EEG) signal processing is shown in Figure 1. First, the input of the model is the EEG time series signal, these electrical signals will convert to point clouds, see Section “From data to point clouds” for details. Second, according to Pearson correlation measure, the adjacency matrix of the nodes coordinate was constructed in this measure space, the construction process is shown in Section “Construction of the adjacency matrix.” Subsequently, the VIPs filtering algorithm is selected to calculate the persistent topology features of the network in Section “Filtering the brain network complexes,” and visualize it as barcodes and persistence diagrams in Section “Visualization of persistent topological features.” Finally, in Section “Stability analysis of persistent topological features,” according to the persistence graph stability theorem, the Bottleneck distance and Wasserstein distance are selected to analyze the stability of persistence features from the aspects of local details and global differences, and then determine the persistence topological features of networks. Section “Experiment and analysis” is the experimental part of the above process.

### From data to point clouds

The preprocessed EEG time series signal is used as the input of the model, and the time series are transformed into points by down-sampling, and then into point clouds after defining the metric space and distance. However, selecting an appropriate distance for translate data points to point clouds is a key issue (Otter et al., 2017). The metric space in EEG data can choose distance metric, correlation metric, or synchronization metric. Scalp electrodes are often used as brain network nodes; however, the collected EEG signals are non-stationary owing to the volume conduction effect, and each node in the network has non-linear dynamic characteristics. Therefore, distance measurement is unsuitable. According to the preliminary experimental work of Zalesky et al. (2012), Pearson correlation is selected to measure the distance between nodes to construct an undirected weighted network. The detailed construction process will be introduced in Section “Construction of the simple complex in schizophrenia task-based data.”

### Construction of the adjacency matrix

The preprocessed EEG time series signal was input into the model and the Pearson correlation measurement space was selected to construct the adjacent matrix for each channel data (i.e., point cloud) of the EEG signal. Based on the characteristics of the EEG signals, an undirected weighted network was constructed, and the electrode channels were taken as network nodes, thus signifying a one-dimensional simplex.

### Filtering the brain network complexes

The process of constructing a nested brain network complex is the process of using PH filtering algorithm to filter. PH is divided into two parts: homology and persistence. Homology in group theory is a tool for classifying topological sets and measuring the specific structure of a simple complex. Persistence is used to extract all given ε persistent structures, that is, to obtain persistent topological features. Among the features, valuable features can be maintained for long durations, whereas noise can be maintained for short durations. This process is called PH. The key steps in constructing the complex are selecting the appropriate filtering threshold ε and filtering algorithm.

#### Selection of the filtering threshold

The selection of the filtering threshold ε is very important (Otter et al., 2017). The common method for this involves selecting different ε values to construct the complex and subsequently finding the ε value corresponding to the effective result. If ε is too small, then the complex may be the original point clouds or several edges of the point clouds. If ε is too large, the original point clouds could form a huge super-dimensional complex.

#### Selection of filtering algorithm

For different practical applications, different types of simple complexes with different attributes must be constructed. Some are easy to describe mathematically and easy to calculate, whereas others are simple but inefficient. For example, some common algorithms for constructing a simple complex are Cech complex algorithm (Espinoza et al., 2020), VIPs algorithm (Choudhary, 2017), alpha algorithm (Jamil and Kim, 2019), and witness algorithm (Guibas and Oudot, 2008). Based on graph filtering, the VIPs complex algorithm is suitable for complex brain networks in complex construction based on graph theory; furthermore, this algorithm has good performance in processing high-dimensional data. Therefore, the VIPs complex algorithm (Zomorodian and Graphics, 2010) was selected for filtering in this experiment.

### Visualization of persistent topological features

When the VIPs filtering algorithm is used to calculate the persistent topological features of the network, with the change in filtering threshold ε, the topological features of the VIPs complex change. During the filtering process, changes in the network topology are visualized using barcodes or persistence diagrams (Carlsson et al., 2005; Ghrist, 2008). The filtering process is used primarily to calculate the *p*-dimensional Betti number interval [ε_*birth*_ε_*death*_], where the ε_*birth*_ is the start time of the *p*-dimensional hole in the simple complex and the ε_*death*_ is the time of its disappearance. Furthermore, they are also the start and end points of the barcode in the barcode’s visualization. These intervals are represented graphically as persistent barcodes, and the persistence diagrams are equivalent to barcodes. In the barcodes, the abscissa represents the time when the persistent features appear, that is, ε_*birth*_; whereas the ordinate represents the time ε_*death*_ when the persistent features disappear. The interval set [ε_*birth*_ε_*death*_] obtained in the filtering process is considered as coordinates of the midpoint of the persistence diagrams, and all pairs of interval sets represent the coordinates to draw the persistence diagrams. The abscissa represents the filtering threshold ε, and the length of [ε_*birth*_ε_*death*_] represents the length of the barcodes. The barcode with a large length represents the persistent topological feature, and the barcode with a short length or only one point represents noise. Correspondingly, the points far from the diagonal represent persistent features, whereas the points close to the diagonal represent noise in the persistence diagrams.

### Stability analysis of persistent topological features

Stability analysis of topological features, i.e., the statistical analysis of barcodes, is a rapidly developing research direction (Lee et al., 2017). This requires the development of corresponding statistical methods and using persistence diagrams to compare and analyze. In the network matching problem, a persistence diagram is created for each network; subsequently, the persistence diagram is compared to obtain the similarity of the network (Agarwal and Sharathkumar, 2014). Currently, three methods can be used to solve the statistical analysis of barcodes (Otter et al., 2017). The first method involves studying the topological features of a random simple complex (Adler et al., 2010; Young et al., 2017). Essentially, when studying the PH, the random simple complex is considered an empty model and the experimental data is compared with it (Stolz, 2014). The second method, which is presently the most common method, involves studying the features of persistence diagrams in specific metric space. The third method involves mapping the space of the persistence diagram to a space suitable for statistical analysis and machine learning methods, such as Banach space (Bubenik, 2015; Bubenik and Dłotko, 2017; Kerber et al., 2017). Such methods include the use of algebraic functions, persistence diagrams and kernel techniques, and persistent landscapes in geometric function space. The second method, which is experiential and suitable for brain network analysis, was selected in this experiment.

The common stability metrics are the Bottleneck distance and Wasserstein distance. A small disturbance to the dataset that causes only a small change in the persistence diagrams before this standard indicates that this is a stable measurement standard.

#### Definition 5

Let *p* ∈ [1,∞), and the *p*-order Wasserstein distance (Zavlanos et al., 2008; Kerber et al., 2017) between two diagrams X and Y is defined as


$$W_{p}\,\left[d\right]\,\left(X,Y\right)=\underset{\emptyset :X\to Y}{\inf}{\left[\sum_{x\,\text{X}}\text{d}\,{\left[\text{x},\emptyset \,\left(\text{x}\right)\right]}^{\text{p}}\right]}^{1/\text{p}},$$

where ∅:X→Y are mappings from X to Y. When *p* = ∞, distance *d* is a measure of a two-dimensional space, and the above formula is expressed as


$$W_{\infty }\,\left[d\right]\,\left(X,Y\right)=\underset{\emptyset :X\to Y}{\inf}\underset{x\in X}{\sup}d\,\left[\left[x,\emptyset \,\left(x\right)\right]\right],$$

where *W*_∞_[*d*_∞_] is the Bottleneck distance (Efrat et al., 2001).

The Bottleneck distance measures the maximum distance between the corresponding matching points of the two diagrams, which can capture large changes of persistence diagrams. The Wasserstein distance measures the total distance between the corresponding matching points of two diagrams, which can provide the overall change in similarity between persistence diagrams. In addition, it is sensitive to small changes in the persistence diagrams.

## Experiment and analysis

### Experimental data and preprocessing

**FIGURE 2:**
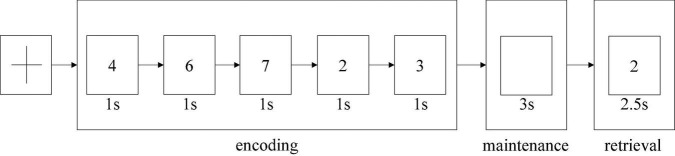
SMST paradigm.

The dataset used in this study was task-based EEG data; it was collected from Beijing Huilongguan Hospital. The experimental paradigm used the modified Sternberg’s short-term memory scanning task (SMST) (Manoach et al., 1999) paradigm (see Figure 2). The experimental processing was divided into three stages, namely, encoding, maintenance, and retrieval.

The Sternberg’s short-term memory scanning task (SMST) paradigm (Sternberg, 1966; Jungeblut et al., 2021) is described in previous studies. Subjects were presented sets of 5 digits from 0 to 9 and were asked to memorize them. In each trial, an initial fixation was presented for 2 s; next, the 5 digits sets were presented for 1 s each (encoding phase) (Figure 2). Following a 3-s delay (maintenance phase), a probe stimulus was presented for 2.5 s (retrieval phase). Participants were asked to indicate whether the probe number was in the previous set of digits by pressing buttons. Probe digits that were present or absent from the encoding phase were presented 50 times each. The reaction time and probe identification accuracy were recorded. Trials in which the reaction time was less than 200 ms were excluded from the analysis.

The subjects came from the Schizophrenia Spectrum Disorder Project of Beijing Huilongguan Hospital. All patients were inpatients of Beijing Huilongguan Hospital and normal controls were recruited from the surrounding community and university. To select proper sample size, we have performed power analysis with G. PowerWin_3.1.9.3 software, and the parameter settings are as follows: effect size | ρ| was 0.5, significance level α was 0.05, power value 1 − β was 0.9. This yielded 34 individuals in per group. So we selected 35 individuals in this experiment. Thirty-five adult inpatients with a Diagnostic and Statistical Manual of Mental Disorders-IV (DSM-IV) (Segal, 2010) diagnosis of schizophrenia were recruited in the present study. Patients with a history of substance abuse within 6 months prior to the date of the experiment or additional neuropsychiatry diagnoses were excluded. Current clinical symptoms were assessed using positive and negative syndrome scale (PANSS) (Kay et al., 1987). An additional 35 normal control participants were recruited from the surrounding community through poster advertisements. Control subjects with a history of mental illness or substance abuse were excluded. There were no significant differences between the two groups with respect to age and sex through using with the double independent sample *t*-test method to compare (Table 1).

**TABLE 1:** Demographic and clinical characteristic of patients with schizophrenia and controls.

Item	Control group	Schizophrenia patients	*p*-value
Numbers	Male:20	Male:13	0.8438
	Female 15	Female 22	
Ages (Years)	37.1 ± 13.8 (21–58)	40.1 ± 11.1 (20–51)	0.3129
Education (years)	11.1 (9–15)	11.4 (9–16)	0.9743
Response time (ms)	1034.9 ± 202.4	1163.3 ± 259.0	0.0001
Response accuracy	0.945 ± 0.076	0.929 ± 0.051	0.0001
PANSS total		52.4 (12.4)	
PANSS positive symptoms		13.3 (5.4)	
PANSS negative symptoms		11.2 (4.2)	
PANSS general symptoms		27.9 (6.2)	

After re-reference, segmentation, removal of eye electric artifacts, and electromyography, the scale of network nodes was 60 and was divided into five bands, namely, θ (4–7 Hz), α (7–14 Hz), β_*1*_ (14–20 Hz), β_*2*_ (20–30 Hz), and γ (30–40 Hz) (Li et al., 2020).

### Construction of the adjacency matrix based on PH

**FIGURE 3:**
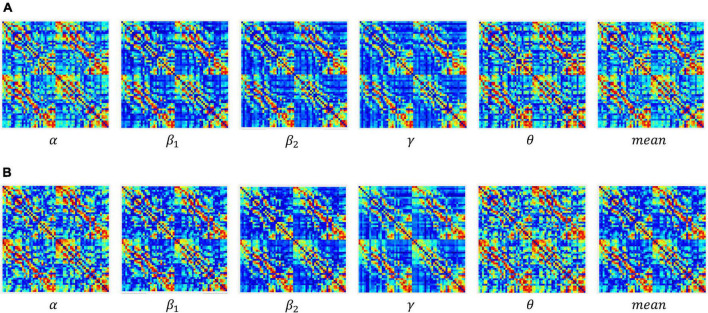
Adjacency matrices at different connection densities in the encoding stage: **(A)** control group, **(B)** schizophrenic patients.

In the experiment, the construction of the adjacency matrix was based on the Pearson correlation (Benesty et al., 2009; Li et al., 2017) metric space, wherein the reciprocal of the Pearson correlation coefficient was taken as the weight of the connection between nodes and an undirected weighted network with dimensions of 60 × 60 was generated. The dynamic adjacency matrix constructed at different sparsity in the coding stage of working memory (WM) in schizophrenia is shown in Figure 3.

**FIGURE 4:**
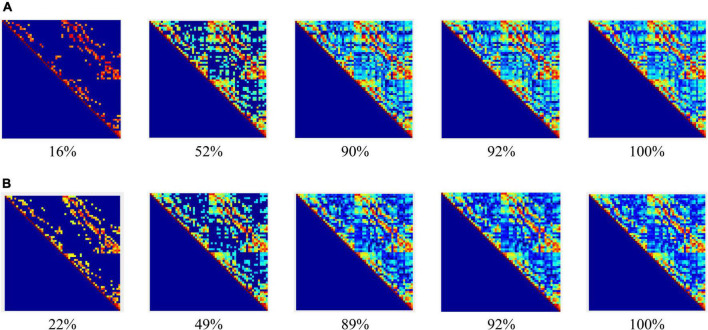
Adjacency matrices at different bands and means in the encoding stage: **(A)** control group, **(B)** schizophrenic patients.

Figure 3 shows that: (1) When the network connection density is small, approximately 20%, there is a significant difference in brain networks between the control group and schizophrenic patients. (2) The change in the connection matrix between the control group and schizophrenic patients gradually decreased from approximately 50% of the network connection density; this indicates that the connection matrix between the control group and schizophrenic patients in the WM coding stage had significantly different features. Moreover, the same result can be observed from the adjacency matrix constructed without the threshold in Figure 4.

### Construction of the simple complex in schizophrenia task-based data

We used the JavaPlex^1^ software package developed by the topology computing group of Stanford University Based on PLEX library. To construct a complex, the following four parameters must be determined. (1) The point clouds coordinate file (.txt), constructed by the edge weight matrix. (2) Maximum filtering threshold ε. (3) Maximum dimension ε_*max*_. (4) Number of filtering steps (*Fs*). These parameters were determined according to the experimental conditions to achieve the best experimental results.

#### Construction of point clouds coordinate file from the edge weight matrix

First, the adjacency matrix was transformed into an edge weight matrix with each row of “*ij*ω_*ij*_.” Next, the high-dimensional matrix that represents the distance between two nodes was mapped to the low-dimensional matrix using the ISOMAP algorithm (Chen et al., 2018). Accordingly, the distance between two points was equal to their distance in the high-dimensional matrix after dimensionality reduction and a group of new sample points were found in the low-dimensional matrix. The ISOMAP algorithm preserves the geometric structure of non-linear data and maintains the global structural information.

**FIGURE 5:**
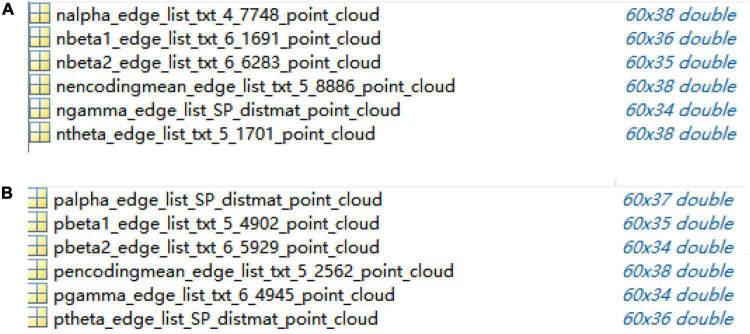
Point clouds from adjacency matrices through ISOMAP algorithm in encoding stage: **(A)** control group, **(B)** schizophrenic patients.

The dimensions of 60 × 60 adjacency matrices of the control group and schizophrenic patients in full band and other five bands were reduced and reconstructed using the ISOMAP algorithm; the results are shown in Figure 5.

#### Maximum filtering threshold ε

After constructing the edge weight matrix, the maximum distance between nodes in each stage was used as the maximum filtering threshold in this experiment. The maximum filtering thresholds in the full band and five bands are listed in Table 2.

**TABLE 2:** Max filtering threshold ε_*max*_.

Band	Control group	Schizophrenic patients
Full band	5.8886	5.2562
θ	5.1701	4.1602
α	4.7748	4.4373
β_*1*_	6.6283	5.4902
β_*2*_	6.628	6.5929
γ	6.2851	6.4945

#### Dimension and number of *Fs*

**FIGURE 6:**
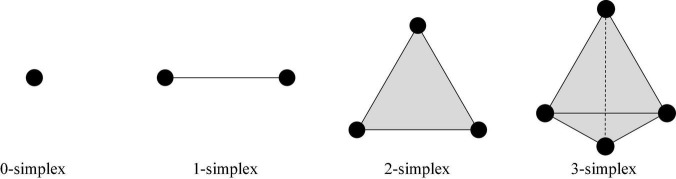
Example of complex.

The complex numbers: running time, dimension0 (*Dim0*), dimension1 (*Dim1*), dimension2 (*Dim2*), and dimension3 (*Dim3*) persistent feature numbers constructed in the three stages were compared. Herein, *Dim0* simplex is the connected components, *Dim1* simplex is the tunnels, *Dim2* simplex is voids, and *Dim3* simplex is three-dimensional triangle. An example of an N-dimensional simplex is shown in Figure 6.

We set the initial value of the maximum dimension to 3; that is, the persistent topological features were extracted in the four dimensions of *Dim0*, *Dim1*, *Dim2*, and *Dim3*. The number of *Fs* was the size of *Fs*. According to literature (Otter et al., 2017), *Fs* is usually set to 20. In this experiment, *Fs* was set as 20, 100, and 1000 to extract the persistent topological features and determine the optimal *Fs* in the model. The experimental results are summarized in Table 3.

**TABLE 3:** Experimental results in three *Fs*.

*Fs*	20	100	1000
Complex numbers	512444	512444	512444
Running time(s)	28.3281	35.9219	37.2188
Numbers of persistent features in *Dim0*	60	60	60
Number of persistent features in *Dim1*	11	13	16
Number of persistent features in *Dim2*	2	2	4
Number of persistent features in Dim3	0	0	0

**FIGURE 7:**
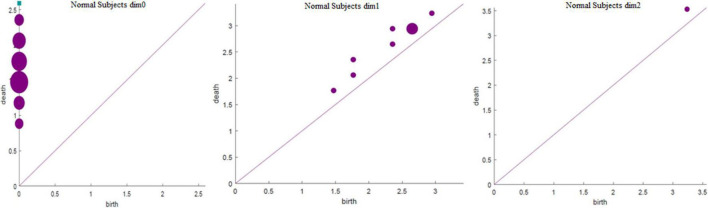
Persistence diagrams (*Fs* = 20) of the coding stage of the control group.

**FIGURE 8:**
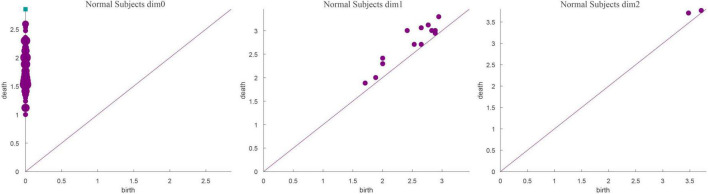
Persistence diagrams (*Fs* = 100) of the coding stage of the control group.

**FIGURE 9:**
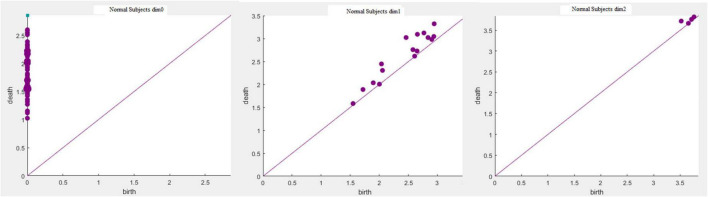
Persistence diagrams (*Fs* = 1000) of the coding stage of the control group.

The running time in Table 3 was obtained using a computer configured as CPU with specifications: Intel (R) core (TM) i7-6700, 32 GB memory, and Windows x64 bit operating system. The data presented in Table 3 shows that the total number of complexes constructed in the three cases remained unchanged. The running time when *Fs* was set as 100 was 21.14% longer than that when *Fs* was set as 20; however, the number of features did not change significantly. In addition, when *Fs* was 1000, the running time was 3.49% more than 100 and the number of features changed significantly. Therefore, to weigh the time efficiency and the number of features, *Fs* can be 20, 100, or 1000 when the amount of data is large. The final *Fs* was determined by visualizing persistence diagrams. Figures 7–9 show the persistent topological features of the control group when *F*s were selected as 20, 100, and 1000.

### Persistent topological features in schizophrenia patients

**FIGURE 10:**
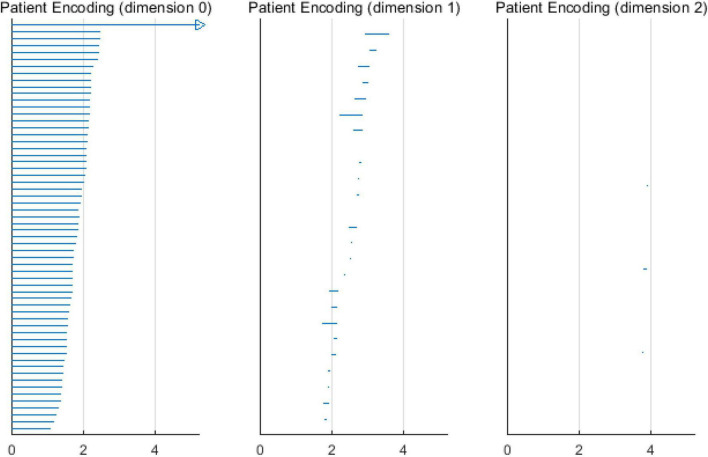
Barcodes of three dimensions in schizophrenic patients coding stage.

**FIGURE 11:**
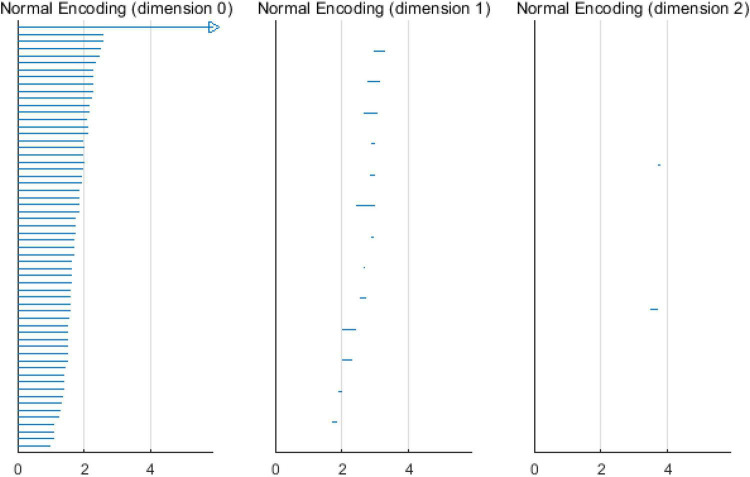
Barcodes of three dimensions in the control group coding stage.

Based on the above experimental conclusions, the optimal parameters are as follows. (1) The maximum dimension was 2. (2) The maximum filtering threshold ε_*max*_ of the control group and schizophrenic patients in each band were the values corresponding to those presented in Table 2. (3) The optimal *Fs* was 20. The persistent features of the brain network in the full band and five bands can be extracted and visualized by barcodes and persistence diagrams, respectively. The barcodes of the full band persistent features of schizophrenic patients and the control group in the coding stage are shown in Figures 10, 11.

Figures 7–9 show that the features of *Dim0* are the same. For *Dim1* and *Dim2*, when *Fs* was selected as 100 and 1000, although the number of features was greater than that when *Fs* was 20, most of the features that were distributed near the diagonal in Figures 8, 9 were noise. Only those that existed in the interval of [3.474274, 3.709818] were persistent topological features. Therefore, the optimal value of *Fs* was 20. Moreover, the data presented in Table 3 shows that the number of features of *Dim3* was always zero; thus, the maximum dimension of filtering was 2.

### Stability analysis of persistent topological features in schizophrenia patients

In this experiment, we used the Bottleneck distance and Wasserstein distance as metrics to compare the persistence diagrams and measure the stability of persistent features. The bottleneck distance and Wasserstein distance were calculated using the GUDHI package in Ripser in the Python environment.

#### Bottleneck distance

The important parameter for calculating the Bottleneck distance is accuracy *e*, which was set as 0.01 to calculate the approximate value; additionally, the same value of 0.01 was set as the default value to calculate the real value. A comparison of the calculation results is presented in Table 4.

**TABLE 4:** Bottleneck distances between persistence diagrams.

Band	*Dim0* real	*Dim0* approximate	*Dim1* real	*Dim1* approximate
Full band	0.53	0.52	0.75	0.71
θ	0.26	0.22	0.21	0.18
α	0.17	0.09	0.22	0.26
β_*1*_	0.30	0.31	0.27	0.35
β_*2*_	0.49	0.46	0.29	0.27
γ	0.63	0.59	0.63	0.08

The data presented in Table 4 shows that except for band α of *Dim0* and band γ of *Dim1*, the errors between the approximate value and real value were very small; further, there may be singular values in the persistent topological features of the two bands.

#### Wasserstein distance

The results of the Wasserstein distance of the *Dim0* and *Dim1* dimensions in the full band and the five bands of the control group and schizophrenic patients are presented in Table 5.

**TABLE 5:** Wasserstein distances between persistence diagrams.

Band	*Dim0*	*Dim1*
Full band	1.54	1.12
θ	0.87	1.44
α	0.83	1.62
β_*1*_	0.76	0.96
β_*2*_	1.98	0.64
γ	2.80	0.59

### Results analysis

In this study, a full-scale complex brain network model was proposed and applied to the WM data analysis of schizophrenic patients, and the related parameters and algorithms of the model were selected according to the experimental analysis. The 60-dimensional matrix was dropped to between 34 and 38 by the ISOMAP algorithm when the edge weight matrix and point clouds file were exchanged, which provided a good foundation for subsequent efficient data processing.

Several important parameters were determined through an all-round experimental effect comparison, listed as follows. (1) The maximum dimension was 2. (2) Experiments showed that there were no persistent topological features in bands β_2_,θ, and γ of the control group, and bands β_*2*_ and γ of schizophrenic patients in *Dim2*. Therefore, the stability analysis required *Dim0* and *Dim1*. (3) The maximum *Fs* was 20, which preserved the important features, improved the time efficiency, and eliminated noise.

For the stability analysis, the results of the Bottleneck distance between persistence diagrams revealed that the approximate value was closer to the real value when the accuracy parameter *e* was the default value, but there was slight difference between the two values when there was no singular value in the persistence diagrams. In addition, the Bottleneck distance in bands α and θ was small; that is, the overall change in the persistence diagrams of the control group and schizophrenic patients was not significant. Therefore, the output of the model can be a persistent topological feature of the two bands.

## Conclusion and future works

In this study, a full-scale brain network analysis model based on PH was proposed. The related algorithm and parameters in the data processing of the model were analyzed and some key problems were investigated, including the construction of nodes and edge weight matrix and the selection of filtering threshold in this network. Furthermore, the model was applied to task-based schizophrenic patients to extract persistent topological features and analyze their stability. The experimental results suggest that the full-scale brain network analysis model can be a stable biological reference standard for stability and noise immunity.

Topological data analysis can extract more hidden topological signal features, which are difficult to be decoded using general signal processing tools. Because the low-dimensional persistent features calculated by VIPs can capture noise (short survival time in barcodes and data points near the diagonal in the persistence diagrams) of the preprocessed data set; hence, the features of persistence diagrams are almost equivalent to those obtained from noiseless data. Therefore, applying PH theory to EEG brain network analysis can solve the problem of threshold selection and noise elimination.

The implicit goal of this study was to extract the topological features of the networks that persist across multiple scales in schizophrenia EEG data. However, there are some limitations to this study. First, the application of the schizophrenia EEG brain network analysis method based on PH theory in large-scale clinical EEG analysis needs further discussion and continuous research. This is required because most of the existing filtering algorithms focus on computational efficiency and cost, and rarely analyze the stability of their applications in large-scale networks. Second, although persistence diagrams are important tool in TDA, the use of machine learning algorithms in the space of persistence diagrams is challenging. One way to resolve this is by transforming the persistent diagram into a vectorized summary, which can be easily used for machine learning tasks. Some alternative representations to persistence diagrams include persistence landscapes (Bubenik, 2015; Vipond, 2020), persistence images (Adams et al., 2017; Som et al., 2020), Betti curves (Giusti et al., 2015; Curto et al., 2021), thermonuclear (Reininghaus et al., 2015), and persistence entropy (Chintakunta et al., 2015; Atienza et al., 2020). Applying these transformed features to popular machine learning methods is another work that our groups are carrying out; herein, persistent topological features are extracted using adaptive thresholding during the persistent homology filtrations. The distribution state of these features are represented by heatmaps and persistence entropies. The process states of persistent feature generation are interpreted by Betti curves and persistence landscapes. Finally, the amount of calculation of high-dimensional data filtered by the *Vietoris-Rips* algorithm is high and increases exponentially. Dynamic determination of the *Vietoris-Rips* filtering threshold ε, which significantly saves calculation time and improves the efficiency of analysis compared with the well-established approaches, will be used in our next study.

## Data availability statement

The original contributions presented in the study are included in the article/supplementary material, further inquiries can be directed to the corresponding authors.

## Ethics statement

The studies involving human participants were reviewed and approved by Beijing Huilongguan Hospital Ethics Committee. The patients/participants provided their written informed consent to participate in this study.

## Author contributions

GG and YZ designed the study. YZ, JH, and ST acquired the data and completed data pre-processing work. CL, XX, and LJ analyzed and interpreted the results of the data analysis. GG and GY drafted the manuscript. YD, YF, DS, and LW revised the manuscript. All authors contributed to the article and approved the submitted version.

## Footnotes

1 JavaPlex[EB/OL]. http://appliedtopology.github.io/javaplex/.
